# Effects of Therapeutic Ultrasound and Aussie Current With High-Intensity Interval Training on Abdominal Adiposity in Young Adults With Overweight and Obesity: Protocol for a Randomized Controlled Trial

**DOI:** 10.2196/71829

**Published:** 2025-07-11

**Authors:** Ana Carolina Aparecida Marcondes-Scalli, Patricia Rehder-Santos, Étore De Favari Signini, Alex Castro, Carla Dato, Leonardo Furlan, Richard Eloin Liebano, Aparecida Maria Catai

**Affiliations:** 1 Cardiovascular Physical Therapy Laboratory, Nucleus of Research in Physical Exercise Universidade Federal de São Carlos São Carlos Brazil; 2 Hospital de Ensino Dr. Washington Antônio de Barros Petrolina Brazil; 3 Biosciences National Laboratory (LNBio) Biosciences National Laboratory (LNBio) Brazilian Center for Research in Energy and Materials Campinas Brazil; 4 Nutrition Course Centro Universitário Central Paulista São Carlos Brazil; 5 Physical Therapy Department Federal University of São Carlos São Carlos Brazil; 6 Department of Rehabilitation Sciences University of Hartford West Hartford, CT United States

**Keywords:** combined therapy, abdominal subcutaneous fat, obesity, lifestyle, physiotherapy

## Abstract

**Background:**

More than half of the world’s population will be overweight or obese by 2035, and it is known that physical exercise, such as high-intensity interval training (HIIT), is a tool for controlling obesity by improving body composition and the metabolic profile. Noninvasive techniques such as therapeutic ultrasound (TUS) and the Aussie current have shown potential in controlling adipose tissue, but their effects combined with HIIT remain unknown. TUS may be combined with the Aussie current to potentiate the specific effects of each intervention, such as lipolysis induced by TUS and lymphatic activation promoted by the Aussie current. The integration of HIIT into this protocol is justified by its ability to stimulate β-oxidation and facilitate the metabolic use of fatty acids mobilized by the electrophysical resources. Furthermore, the use of HIIT as opposed to moderate-intensity continuous training contributes to reducing the total duration of the intervention.

**Objective:**

This study aims to evaluate the effects of TUS+Aussie current combined with HIIT on body composition, serum metabolic profile, and cardiovascular autonomic modulation (CAM) in individuals with overweight and obesity.

**Methods:**

This is a randomized, double-blind (researcher and outcome assessor) clinical study. The participants will be randomized into 3 groups: active TUS+Aussie current with HIIT group, placebo for TUS+Aussie current with HIIT group, and TUS+Aussie current–only group. All participants will undergo nutritional monitoring 30 days before the proposed interventions to adjust macronutrients, optimize energy intake, and improve diet quality. Primary outcomes include changes in subcutaneous adipose tissue thickness, body composition, and serum metabolic profile. Secondary outcomes assess perceived stress, body image, blood biochemistry, sleep quality, and CAM. Data analysis involves linear mixed models estimated using the maximum likelihood method with an appropriate covariance matrix structure.

**Results:**

A total of 60 participants will be recruited and randomized between February 2024 and June 2025. The baseline assessments and intervention are scheduled to be completed in August 2025, and data collection will be completed by the end of September 2025. Data acquisition is still ongoing; therefore, data analysis has not yet been carried out.

**Conclusions:**

This is the first study to combine TUS+Aussie current with HIIT, potentially integrating the effects of lipolysis and fat oxidation and possible changes in the serum metabolic profile and CAM. The results could optimize treatment duration, promote changes in lipid profile, and maintain cardiovascular health in people with overweight and obesity.

**Trial Registration:**

Brazilian Registry of Clinical Trials RBR-4xh6232, Universal Trial Number: U1111-1287-2345; https://ensaiosclinicos.gov.br/rg/RBR-4xh6232

**International Registered Report Identifier (IRRID):**

DERR1-10.2196/71829

## Introduction

### Background

According to a report by the World Obesity Federation, more than half of the world’s population will be overweight or obese by 2035 [1]. Excess abdominal fat is a primary determinant for developing insulin resistance and other metabolic disorders [2]. Lifestyle modification is the most recommended approach for treating and preventing obesity at any stage of life [2]. In addition to exercise and a controlled diet, noninvasive techniques have emerged as safe and effective tools for managing adipose tissue [3,4]. These techniques include different electrophysical agents, such as low-intensity laser [5], cryotherapy [6], radiofrequency [7], infrared radiation [8], and therapeutic ultrasound (TUS) [4,9].

TUS is a therapeutic modality widely used in physiotherapy clinical practice [10,11]. The nonlinear propagation of TUS waves induces mechanical forces on particles at the applied site, resulting in biological effects [4], including pyroptosis [12]. This term refers to a form of programmed cell death mediated by caspase-1 or caspase-11, characterized by the secretion of proinflammatory cytokines [12,13]. Cells undergoing pyroptosis exhibit mitochondrial membrane potential loss; DNA fragmentation; nuclear condensation; and the release of cytoplasmic contents into the bloodstream, including fatty acids [13,14].

TUS can be combined with Aussie electrical current to stimulate the lymphatic system, producing a strong but comfortable paresthesia [11,15]. The Aussie current is characterized by a medium-frequency alternating wave modulated at a low frequency (LF) and is indicated for various applications, including muscle strengthening, pain control, lymphatic drainage, and edema control [11]. The combined effects of these electrophysical resources—TUS and Aussie current—alongside aerobic exercise after therapy may enhance fat β-oxidation. However, despite promising results for cellulite reduction with the combination of TUS and Aussie current [16-18], previous studies are limited by small sample sizes; lack of a placebo group; and insufficient focus on posttherapy clinical outcomes, such as serum metabolic changes [18]. In addition, other forms of intervention have been widely suggested for reducing body composition and promoting significant metabolic changes in individuals with obesity, such as high-intensity interval training (HIIT). HIIT alternates between periods of high intensity and active recovery and has become a popular training method due to its time efficiency.

Although previous studies [19,20] have shown better effects of HIIT on the aerobic capacity of patients with cardiovascular disease when compared to continuous moderate-intensity training, the effects of HIIT on cardiovascular autonomic modulation (CAM) in people with obesity are poorly understood. A systematic review evaluated the effects of HIIT on CAM based on 6 studies that used heart rate (HR) variability as a measure [21]. The results showed that HIIT can improve autonomic modulation by increasing parasympathetic cardiac modulation and reducing sympathetic cardiac modulation in healthy individuals and patients with metabolic syndrome, making it a promising strategy for cardiovascular rehabilitation [21].

As for the effects of HIIT on body composition, another review analyzed body fat percentage, fat mass, and fat-free mass in cycling, running on the ground, and running on a treadmill [22]. The findings showed that all forms of HIIT significantly reduced fat mass (−1.86 kg) and body fat percentage (−1.53%) compared to the control group [22].

Although previous studies have reported reductions in abdominal circumference and body composition following TUS and Aussie current application [3,4,10,11], none have evaluated this technique in combination with HIIT in participants with overweight or obesity. In addition to optimizing time, the combination of both interventions also aims to optimize the breakdown, drainage, and oxidation of fat, promoting weight loss. There is a need for clinical trials to investigate the potential clinical effects of this therapeutic modality in humans, as suggested by animal models [16-18]. Furthermore, this study addresses a relevant public health problem [1]—the increasing prevalence of obesity in young adults—by proposing a multimodal, noninvasive intervention aimed at reducing abdominal adiposity and improving metabolic health, a scope that has been explored little in previous clinical protocols [4,9,11]. The results of this study will determine whether TUS and Aussie current therapy induce changes in body composition, manage serum metabolic profile, and improve CAM indexes.

### Objectives

This pragmatic study aims to evaluate the effects of combined therapy (TUS+Aussie current) alone and combined with HIIT on body composition, serum metabolic profile, and CAM in people with overweight and obesity. This study also investigates the impact of these interventions on perceived stress, body image, and sleep quality in people with overweight and obesity.

## Methods

### Study Design

This is a randomized, pragmatic, placebo-controlled, double-blinded, 3-arm (1:1:1) clinical trial. The protocol was developed in accordance with the SPIRIT (Standard Protocol Items: Recommendations for Interventional Trials) guidelines [23].

This research will be conducted at the Federal University of São Carlos (UFSCar) in the Cardiovascular Physical Therapy Laboratory/Physical Exercise Research Center within the Department of Physical Therapy. Participants will be recruited through social media advertisements. An electronic form hosted on Google Forms will be shared via Instagram. Interested participants who meet the basic inclusion criteria will be contacted via WhatsApp to provide additional information about the study and assess their eligibility. Participants who do not respond to the messages will receive up to 3 phone call attempts at different times. If these contact attempts are unsuccessful, the individual will be considered unreachable and ineligible for the study.

### Ethical Considerations

This study adheres to the ethical principles outlined in the Declaration of Helsinki. The research protocol was approved by the Research Ethics Committee of the UFSCar (approval 64624522.8.0000.5504) and was registered in the Brazilian Registry of Clinical Trials on February 8, 2023, and updated on December 2, 2024 (RBR-4xh6232—Universal Trial Number: U1111-1287-2345). Participants will be informed of their right to withdraw from the study at any time without penalty or loss of benefits. In addition, all collected data will be treated with strict confidentiality and used solely for research purposes. This is an academic study and no compensation will be given to participants.

Before any data collection, the outcome assessor obtains written informed consent from participants. Only those who provide consent proceed to the baseline evaluation and randomization.

The data will be collected on paper and stored in folders at the Cardiovascular Physical Therapy Laboratory (Federal University of São Carlos). The data will be entered into Microsoft Excel (Microsoft Corp) by the physiotherapist responsible for the assessments, reassessments, and follow-ups. In addition, all the data will be available on a public research bench.

The database and electronic analyses will be stored on a secure computer server, with personal log-in access authorized by the principal investigator of this study. The principal investigator will have access to the complete dataset (without the allocation groups), and the data and materials in this paper will be available to other researchers on request. Upon completion of the study, all data and documents will be archived by the principal investigator for 5 years at the Physical Therapy Department of the Federal University of São Carlos.

### Eligibility Criteria

The eligibility criteria for this study are outlined in Textbox 1.

**Inclusion and exclusion criteria.**

**Inclusion criteria**

- Men and women
- Ages of 18 to 40 years at enrollment
- Classified as overweight or having grade-I or grade-II obesity according to the fat mass index as measured using dual-energy x-ray absorptiometry
- Healthy adults without any chronic or acute disease and who started any physical activity in the previous 3 months

**Exclusion criteria**

- Current smokers, individuals with chronic alcohol misuse, illicit substance users, or smokers or ex-smokers less than 12 months old
- History of body mass reduction treatments using drugs or electrophysical agents such as therapeutic ultrasound and electrotherapy in the previous 12 months
- Electrocardiogram abnormalities, including ischemia, overloads, conduction disorders, and serious arrhythmias such as ventricular tachycardia
- Presence of any of the following conditions or diagnoses of diseases: neurological, renal, respiratory, cardiac, or musculoskeletal conditions; venous thrombosis; anticoagulant therapy or bleeding disorders; active cancer or cancer treatment in the previous 5 years; conditions or lesions in the abdominal region; diabetes mellitus type 1 or 2; hypothyroidism or hyperthyroidism; and pregnancy in the previous 12 months

### Randomization and Blinding

In this study, the researchers responsible for the evaluations and those analyzing the data will be blinded. To ensure the feasibility of blinding, the blinded research teams will not participate in the interventions, and all information will be coded to prevent the identification of participants and intervention groups. The effectiveness of blinding will be assessed using a questionnaire completed by the evaluators at the end of the final assessment for each data collection block.

After the initial assessments, participants will be stratified (age range, sex, and fat mass index) and randomly assigned to 1 of 3 groups. Participants in group 1 (active TUS+Aussie current with HIIT [AG]) will undergo combined therapy (TUS+Aussie current) along with the HIIT protocol. Participants in group 2 (placebo for TUS+Aussie current with HIIT [PG]) will undergo placebo combined therapy (TUS+Aussie current) with the devices turned off along with the HIIT protocol. Participants in group 3 (TUS+Aussie current–only group [HG]) will receive only the combined therapy (TUS+Aussie current) without the HIIT protocol.

For stratified randomization, participants will be initially grouped into blocks based on similar characteristics, including age range (20-29 years or 30-40 years), sex (male or female), and fat mass index (overweight, grade-I obesity, or grade-II obesity). These characteristics will be chosen to control for potential confounding factors. Within each block, participants will be then randomized into the 3 study groups using Microsoft Excel, ensuring an unbiased allocation process. Block sizes were not fixed, further reducing the risk of allocation predictability. The randomization process will be conducted by a researcher designated exclusively for this task, who will have no contact with the study participants or the researchers involved in data collection. This researcher will also be responsible for generating random alphanumeric codes to ensure the anonymity of each participant’s information.

### Measures

#### Baseline Evaluation

Participants who meet the eligibility criteria will be considered eligible for randomization and will have a baseline evaluation scheduled. To characterize the sample, the initial assessment records information, such as sex, age, anthropometric data, family history, medication use, educational level, occupation, and comorbidities.

#### Study Assessments and Timeline

All study participants will have to attend 2 face-to-face visits: one at baseline (week –1) and one at the final assessment (week 6). In addition, participants will be assessed by a nutritionist and receive nutritional guidance and diet plans (week 0). The treatment will last 5 weeks. A detailed schedule is available in Table 1.

**Table 1:** Study period.

Time point^a^		Study period								
		Enrollment	Allocation	After allocation						Closeout
		Week −4	Week 0	Week 1	Week 2	Week 3	Week 4	Week 5	Week 6	
**Enrollment**										
	Eligibility screening	✓								
	Informed consent	✓								
	Nutritional evaluation	✓								
	Allocation		✓							
**Interventions**										
	AG^b^			✓	✓	✓	✓	✓		
	PG^c^			✓	✓	✓	✓	✓		
	HG^d^			✓	✓	✓	✓	✓		
**Assessments**										
	SAT^e^	✓	✓						✓	
	Body composition	✓	✓						✓	
	Metabolic profile			✓				✓		
	Perimetry	✓	✓						✓	
	Blood test	✓	✓						✓	
	Questionnaires	✓	✓						✓	
	Exercise test		✓							
	24-h food recall			✓	✓	✓	✓	✓		
	CAM^f^	✓	✓	✓				✓	✓	

^a^Timepoint: evaluation time.

^b^AG: active therapeutic ultrasound+Aussie current with high-intensity interval training group.

^c^PG: placebo for therapeutic ultrasound+Aussie current with high-intensity interval training group.

^d^HG: therapeutic ultrasound+Aussie current–only group.

^e^SAT: subcutaneous adipose tissue.

^f^CAM: cardiovascular autonomic modulation.

#### Initial Evaluation

Initial information (lifestyle and inclusion criteria) will be collected online using a screening form link for men and women. Body mass and height measurements will be taken using a calibrated scale. Participants will be advised to be barefoot and wear light clothing. Height is measured with the participant standing and using a stadiometer. A member of the research team (ACAMS) is responsible for carrying out all study assessments as well as screening and recruiting participants, minimizing bias.

#### Abdominal Subcutaneous Adipose Tissue

Ultrasound will be used as the main end point to assess abdominal subcutaneous adipose tissue (SAT) [4]. Intrarater reliability was assessed in previous studies [24]. A GE HealthCare Venue 40 ultrasound (model NZCART; GE Medical Systems, Ltd) is used. Subcutaneous fat, defined as the distance in centimeters between the dermis and the external surface of the fascia of the abdominal muscles, is measured using a linear transducer with a frequency of 12 MHz placed transversely 1 cm above and 1 cm below the umbilical scar [25,26]. Participants will be examined in the supine position without any artifact compressing the abdominal region, with their knees bent, and instructed to breathe in and out. A total of 3 measurements will be taken, each at the end of an exhalation, with minimal pressure on the abdominal cavity for greater measurement precision [27,28].

#### Body Composition

To determine total body mass, fat mass percentage per region, and visceral fat, we use dual-energy x-ray absorptiometry (DXA; Hologic Discovery A). This assessment is considered the gold standard for evaluating body composition in individuals with overweight and obesity [29]. In addition, DXA is used to classify participants as overweight or obese (grade I and II) [30]. Considering that hormonal variation throughout the menstrual cycle can influence body composition results, especially through fluid retention and water redistribution, all female participants will be assessed during the follicular phase of the menstrual cycle, a phase in which there is greater hormonal stability and a reduction in extracellular fluids, providing greater accuracy in body composition measurements [31].

Participants must fast for 4 hours, refrain from vigorous exercise for at least 12 hours, avoid caffeine and alcohol for the previous 24 hours, and consume a normal meal the night before the test. They should not wear any metal objects or accessories during the test to prevent interference with the total body composition results. The participant is placed in a supine position with internal rotation of the thighs, legs, and feet and instructed not to speak or move during the examination. The results obtained will be transmitted to a computer connected to the equipment, where the report shows the data in grams and percentages and which will be used to analyze total lean mass (kg), total bone mass (percentage), total fat mass (g), fat mass index (kg/m^2^), fat mass in the android and gynoid regions (g), and visceral fat (g) and, through calculations, deduce subcutaneous fat [32,33].

#### Serum Metabolic Profile

Venous blood samples will be collected in the cubital fossa by a specialized professional after the participants have fasted for 3 hours following a standardized meal. Blood samples will be taken 3 times: before and immediately after the TUS+Aussie current session and immediately after HIIT. Each tube of blood collected will be left to stand at room temperature for 30 minutes. They will then be centrifuged at 1450 × g for 10 minutes (Sorvall ST 8 Benchtop Centrifuge; Thermo Scientific). The serum is divided into 3 aliquots in 1.5-mL microtubes using 5-mL disposable pipettes and stored immediately at −80 °C until further analysis. The serum samples will then be thawed at the UFSCar Department of Chemistry and sent for analysis using the hydrogen nuclear magnetic resonance technique. Serum glycerol, total cholesterol, very low–density lipoprotein, low-density lipoprotein (LDL), high-density lipoprotein, and triglycerides will be assessed. These will be measured using wet chemistry (except for LDL, which will be calculated using the Friedwald equation; ADVIA 1800; Siemens). The experimental procedures for this analysis will follow those described in the work by De Favari Signini et al [34] and Castro et al [35].

#### CAM Measurement

To ensure the quality of the electrocardiogram (ECG) signal collection, the recommendations suggested by Catai et al [36] will be followed. All participants will be instructed to have a regular night’s sleep the night before, avoid alcoholic and caffeinated drinks, refrain from strenuous physical exercise at least 24 hours before and on the day of the assessment, and avoid heavy meals up to 2 hours before the assessment. All procedures will be explained beforehand to familiarize participants with the equipment and the assessor. The experiment will be carried out in the afternoon in a quiet room with the minimum number of people in the Cardiovascular Physical Therapy Laboratory, with controlled temperature and relative humidity (22-23 °C and 40%-60%, respectively) [36]. Before starting data collection, the participants will rest for 10 to 15 minutes to stabilize the signals [36]. After this, they will remain in the supine position for 15 minutes and then perform active postural change, remaining in the orthostatic position for 15 minutes. Participants will be instructed to breathe spontaneously and not to move or speak during the experiment.

RR intervals will be captured via ECG (Bio Amp FE132; ADInstruments) from the modified shunt in the fifth left intercostal space lead. In addition, respiratory movements will be captured using a respiratory belt positioned around the participants’ chest (Marazza) to obtain the respiratory rate during the test. Arterial pressure (AP) will be measured continuously beat to beat using a photoplethysmograph (Finometer PRO; Finapres Medical Systems) on the middle finger of the right hand. RR interval signals, AP, and respiratory movement will be sampled at 1 kHz using a commercial acquisition device (PowerLab 8/35; ADInstruments).

Classic linear indexes of HR variability will be calculated in the time and frequency domains. The indexes calculated in the time domain will be the SD of the NN interval, which represents sympathetic and parasympathetic modulation together, and the square root of the mean of successive NN intervals, which indicates parasympathetic modulation [37,38]. After detecting the QRS complex on the ECG, the apex of the R wave will be identified using parabolic interpolation. The heart period (HP) will be calculated as the temporal distance between 2 consecutive parabolic apexes. A stable sequence of 256 beats (for systolic AP [SAP] and HP) will be chosen in the supine and orthostatic positions, and if isolated ectopic beats are present, they will be linearly interpolated [39]. From these 256 points, the mean and variance of the HP and the mean and variance of the SAP will be calculated in the time domain.

The variability parameters of the HP and SAP will be evaluated according to the autoregressive model [39]. The spectral components will be broken down into LF (from 0.04 to 0.15 Hz) and high frequency (>0.15 to 0.4 Hz) reported in absolute units. The power of the spectral components will be expressed in absolute units or normalized units [38].

Symbolic analysis will be used to evaluate the HP and SAP series as defined in the study by Porta et al [40]. This technique is based on the 6-level uniform quantization procedure applied to HP and SAP series, which transforms series into sequences of symbols (from 0 to 5) from which patterns of 3 consecutive symbols will be constructed [41]. All possible patterns will be grouped without loss of information into families according to the number and type of variations between subsequent symbols: (1) 0V pattern—all symbols are the same; (2) 1V pattern—2 underlying symbols are the same, and the rest are different; (3) 2LV pattern—the 2 variations between adjacent symbols have the same sign; and (4) 2UV pattern—the 2 variations between adjacent symbols have opposite signs. Percentages of patterns within each experimental session were evaluated and denoted using 0V%, 1V%, 2LV%, and 2UV%, respectively. The 0V% index will be understood as a marker of sympathetic modulation when calculated in the HP and SAP series, and the 2UV% index will be understood as an index of vagal modulation when calculated in the HP series [40,42,43].

The baroreflex system will be evaluated through cross-spectral analysis using a bivariate autoregressive parametric approach [44]. The time-series relationships between AP and HP will be represented as coherence, phase, and transfer function gain. The coherence function shows the degree of association between the HP and SAP variabilities [44,45]. Its values range from 0 to 1, where values closer to 1 represent better coupling between the signals. The phase function represents the temporal relationship between the series (ie, the delay between the change in the SAP signal resulting from the heartbeat) and will be evaluated in high frequency and LF [44].

#### Sleep Quality

Sleep quality is assessed using the Pittsburgh Sleep Quality Index for sleep quality, adapted for Brazilians by Bertolazi et al [46]. It is a reliable instrument (intraclass correlation coefficient=0.65) [47] that assesses 7 components of sleep: subjective quality, sleep latency, sleep duration, sleep efficiency, sleep disturbances, medication use, and daily dysfunction. For each component, the score ranges from 0 to 3, and the sum yields a maximum score of 21. Scores of >5 indicate poor sleep quality.

#### Anxiety, Depression, and Stress

Anxiety and depression will be assessed using the Hospital Anxiety and Depression Scale validated for Brazilians [48]. It consists of 14 items divided into 2 domains (depression and anxiety) with 7 items each. The items are scored on a 4-point Likert scale (from 0 to 3), resulting in total scores ranging from 0 to 21 for anxiety and depression. The cutoff points indicating moderate to severe symptoms are ≥8 for the anxiety domain and ≥9 for the depression domain.

Stress is assessed using the Depression, Anxiety, and Stress Scale, which measures the levels of these disorders based on behaviors in the previous 7 days. The items use a 21-question Likert scale, which has been translated into Brazilian Portuguese, to rate the frequency or severity of the participants’ experiences on a 4-point scale [48].

#### Body Image

The Body Shape Questionnaire, validated for the Brazilian population [49], is a self-administered questionnaire consisting of 34 items designed to measure satisfaction and concerns about body shape. It is organized on a 6-point scale: 1=*never*, 2=*rarely*, 3=*sometimes*, 4=*frequently*, 5=*very frequently*, and 6=*always*. The score is the sum of the answers, where ≤110 points indicates no concern, ≥111 to ≤138 points indicates mild concern, ≥139 to ≤167 points indicates moderate concern, and >168 points indicates serious concern [49]. This questionnaire helps assess potential changes in satisfaction or concern about the body after the proposed treatment.

#### Biochemical Blood Tests

Participants will be referred to a specialized clinic for blood collection. They will be instructed to fast for 10 hours before the test. The following will be collected: complete blood count, glycated hemoglobin (through high-performance liquid chromatography), fasting glycemia, insulin resistance (through enzymatic and chemiluminescent immunoassay), evaluation of the homeostatic model, C-reactive protein (through immunoturbidimetry), homocysteine (through chemiluminescence), total cholesterol, high-density lipoproteins, and LDLs and triglycerides (through the enzymatic method). The tests will be clinically relevant in the context of obesity and abdominal adiposity and may contribute to future studies to determine whether any changes occur in these variables after the intervention. The results will be reported by the unit’s physician and sent to the researcher.

#### Perimetry

The waist-to-hip ratio is a calculation based on waist and hip circumference measurements [50]. It is often used to assess the risk of developing diseases such as high cholesterol, diabetes, and high blood pressure [50]. The presence of these cardiovascular risk factors, associated with increased abdominal fat, leads to a high risk of diseases such as myocardial infarction, stroke, and hepatic steatosis and mortality. The values considered normal for male and female individuals are <0.95 and <0.80 cm, respectively [50]. The waist-to-height ratio, on the other hand, is an index that assesses health risks by indicating the proportionality between waist circumference (a possible indicator of central fat accumulation) and height, suggesting a risk of cardiovascular or metabolic diseases [51]. Values of <0.5 cm indicate a lower risk of diseases, whereas values of >0.5 cm indicate a higher risk [51]. The measurements will be taken with the participant standing upright wearing light clothing. The waist circumference is measured as the average distance between the last floating rib and the anterior superior iliac crest [52]. The hip circumference is measured around the widest part of the buttocks at the level of the greater trochanter of the femur [52]. All measurements will be taken twice using an inextensible tape measure, and if there is a difference of 3 cm, a third measurement is taken for both waist and hips [52]. The ratio between the measurements is calculated as the waist circumference divided by the hip circumference.

Neck circumference is measured using an inextensible tape measure perpendicular to the longitudinal axis of the neck over the thyroid cartilage [53]. Participants will be in an anatomical position, either standing or sitting, with their head in the Frankfurt plane and their shoulders relaxed and in inspiratory apnea [53]. The cutoff points for overweight and obesity will be 37 cm for men and 34 cm for women [54]. The values above these cutoffs indicate increased cardiovascular risk factors [54].

#### Physical Activity

All participants will be characterized by their level of physical activity at a single point in time (before the intervention). The long version of the International Physical Activity Questionnaire is used. The International Physical Activity Questionnaire is a validated instrument for the Brazilian population that assesses the level of physical activity. It has 27 questions related to physical activities performed in a week at different intensities—light, moderate, and vigorous—lasting at least 10 minutes continuously. The questions cover 4 dimensions: work, transportation, domestic activities, and leisure, as well as the time spent seated [55]. The score is calculated by adding the workload for each subitem separately [55]. This questionnaire helps classify the level of physical activity before the proposed interventions.

Before the intervention, a treadmill ergometric test is conducted using the Ellestad protocol [56] for clinical cardiological assessment and to assist in prescribing HIIT. The test is interrupted according to the criteria recommended by the third version of the Sociedade Brasileira de Cardiologia ergometry guidelines [57]. The original Borg scale is used to assess the participants’ perception of dyspnea, lower-limb fatigue, and angina during exercise. This scale ranges from 0 to 10, where 0 is no dyspnea and 10 is the most intense dyspnea, fatigue, and angina [58]. The following determines the presence of maximum or peak effort: peak HR (HR_peak_)≥85% of that predicted for age (220 – age) and Borg perceived exertion of 18 on a scale from 6 to 20 (patient exhausted) [59].

### Interventions

#### Nutritional Intervention

The nutritional intervention will be conducted by an experienced nutritionist. All participants receive nutritional guidance 30 days before the combined therapy and HIIT. This initial intervention aims to adjust macronutrient intake, optimize energy consumption, and improve diet quality by reducing the consumption of ultraprocessed foods and increasing the consumption of minimally processed foods. Macronutrient adjustments follow the recommendations of the dietary reference intakes, with carbohydrates comprising 45% to 65%, proteins comprising 10% to 35%, and lipids comprising 20% to 30% of the total caloric intake [60].

Following this phase, participants undergo a new series of assessments and then begin 5 weeks of combined therapy and HIIT. During these 5 weeks, dietary control is monitored by the nutritionist to ensure consistency in dietary patterns throughout the study. Daily energy intake is considered stable if variations remain at <10% during the protocol.

Food intake will be assessed weekly using a 24-hour recall, a questionnaire that quantifies all food and drink consumed in the previous 24 hours. Participants will receive an electronic link once a week during the 5-week intervention. The data collected will be analyzed by a nutritionist to assess consistency and adherence in total energy and macronutrient intake. Variations of >10% in total calorie intake will be discouraged to ensure dietary stability throughout the protocol. No additional methods of validating adherence to the diet will be used as the weekly recalls provide a feasible and easy monitoring strategy for the participants. Total energy intake and macronutrient composition will be calculated using the Dietbox program [61]. Energy requirements will be estimated based on the dietary reference intakes [62]. For qualitative analysis, foods will be categorized according to their processing level—natural, minimally processed, culinary ingredients, processed, and ultraprocessed—as defined by the 2014 Food Guide for the Brazilian Population [63].

#### Combined Therapy Application Time Calculation

Before the first session, the application time for the combined therapy will be individually determined. Measurements will be conducted by the same blinded assessor using an inelastic tape measure with the participant in a standing position. Specifically, the horizontal distance between the last floating ribs (x) and the vertical distance to the midpoint between the anteroinferior iliac crests (y) will be measured. The abdominal area (cm^2^) will be calculated by multiplying these 2 values (x × y) and then dividing the result by 18. This divisor corresponds to the total effective radiating area of the ultrasound head used in the therapy, which consists of 3 spheres, each with an effective radiating area of 6 cm^2^. Dividing the abdominal area by 18 allows us to determine the time required to apply the therapy to each participant according to the following formula: application time = (x × y)/18.

For example, if a participant has a horizontal distance (x) of 21 cm and a vertical distance (y) of 26 cm, the area will be calculated as follows: 21 × 26 = 546 cm^2^. Dividing this area by 18 results in approximately 30 minutes of application time (546/18 = 30.33 minutes).

#### Pretraining Instructions

Before each training session, participants will be instructed to (1) wear comfortable clothing and exercise-appropriate footwear, (2) ensure adequate sleep the previous night, (3) hydrate with water 2 hours before the session and bring a personal water bottle, and (4) avoid strenuous physical activity and alcoholic consumption within 24 hours before the session.

Food consumption will also be recommended 24 hours before the first and last sessions, as described in the Nutritional Intervention section.

#### Session Scheduling and Follow-Up

To minimize data loss, participants receive reminders about the date and time of each session. Physiotherapists responsible for interventions may also call participants if necessary to confirm appointments. For evaluations and re-evaluations, the blinded evaluator contacts the participants via telephone. At the end of the 10 treatment sessions, the re-evaluation is scheduled between 3 and 15 days after the last session.

### Group 1: AG

Participants in the AG will be treated with TUS+Aussie current (Heccus; Ibramed). All therapists who will administer the TUS+Aussie current interventions have undergone standardized previous training, which includes theoretical and practical sessions to ensure the fidelity of the protocol. In addition, every participant will be seen by each therapist at least once. This strategy aims to minimize possible biases related to therapist application and intervention quality in all groups.

The protocol combines continuous ultrasound (3 MHz; 2 W/cm^2^) and Aussie current (1 kHz; modulated at 10 Hz). The participant will be instructed that the sensation must be tingling and comfortable (sufficient intensity to produce strong but comfortable paresthesia without stimulation to painful paresthesia) [64]. In every session, and every 5 minutes, the therapist will record the intensity of the Aussie current and evaluate the levels of paresthesia on a scale from 0 to 10, with 0=no tingling sensation and 10=maximum tolerable tingling. Given the possible habituation to the paresthesia caused by the Aussie current, the scale will be applied every 5 minutes during the session, and a score of 5 will be set as the threshold for adjusting the intensity of the current (ie, tolerable or supportable tingling). The target intensity is set at approximately 5. The intensity of the Aussie current will be adjusted immediately whenever the perception reported by the participant falls to <5 (0-10), ensuring a consistent and comfortable sensory experience throughout the session.

The treatment is applied to the abdominal region using ultrasound. The therapist ensures consistent pressure and maintains slow, slightly circular, horizontal movements without losing contact with the surface.

In the final session, participants complete a printed questionnaire that includes the reported intensity of the Aussie current and the following questions: “Would you recommend this therapy to someone else?” and “Do you believe you received the active treatment, the placebo or are unsure? Why?” In addition, in the last session, the blinded therapist will answer a questionnaire about which group they believe each participant was allocated to and the reason for this choice.

Immediately after the TUS+Aussie current application, participants will take to the treadmill for the HIIT session. Participants will be instructed to maintain proper posture with relaxed shoulders, a forward-facing stance, and a breathing pattern of inhaling through the nose and exhaling through the mouth, avoiding apnea during the exercise. The Borg scale (6-20) [65] will be applied 10 seconds before each load change during the 20-minute interval protocol.

HR is continuously monitored using a Polar H10 HR monitor attached to an elastic chest strap positioned at the xiphoid process. This Polar monitor is paired via Bluetooth with a tablet.

**Figure 1:**
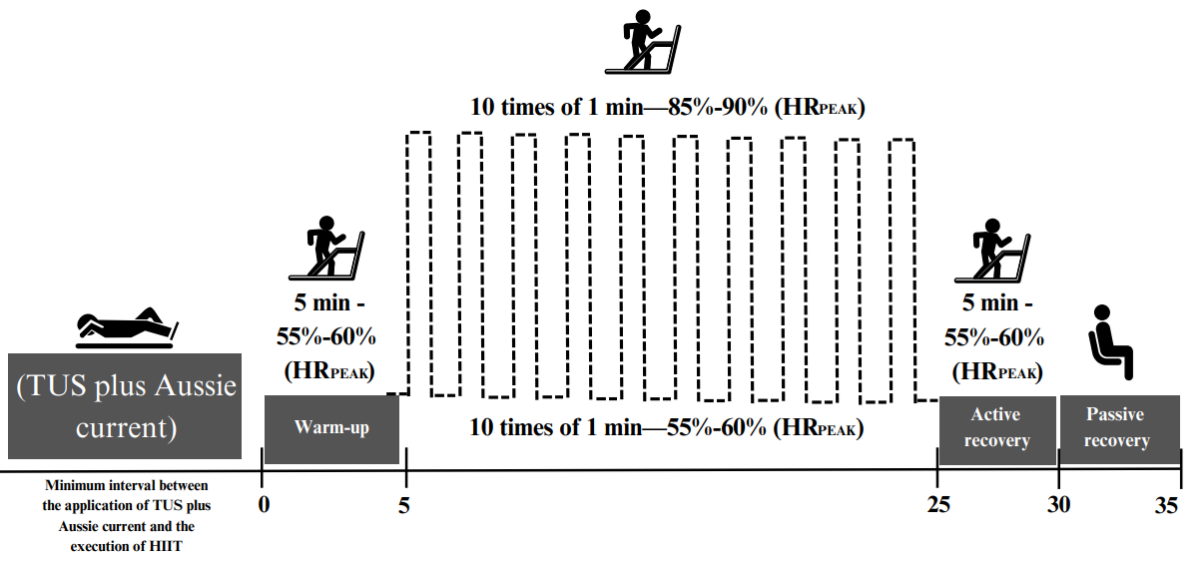
Summary of the high-intensity interval training (HIIT) protocol following the application of combined therapy, along with the progression of the intervention protocol. HRpeak: peak heart rate during the ergometric test; TUS: therapeutic ultrasound.

The HIIT protocol lasts 30 minutes and includes the following:

1. A warm-up of 5 minutes, progressively reaching 55% to 60% of the peak HR determined in the ergometric test.
2. An interval phase of 10 cycles of a 1-minute high-intensity interval (85%-90% of peak HR) interspersed with 10 cycles of a 1-minute light- to moderate-intensity interval (55%-60% of peak HR). After 5 sessions, the load will be updated by 5% to 10% considering physiological adaptations (moderate intensity 60%-65% of peak HRand high intensity 90%-95% of peak HR). After the fifth training session, the training load will be adjusted by 5% to 10% based on physiological (peak HR) and subjective (Borg) criteria. Specifically, participants who consistently reach the target HR zone (85%-90% of peak HR) during high-intensity intervals and report a perceived exertion score of <17 on the Borg scale (6-20) will have the speed or incline of the treadmill increased by 5% to 10%. HR will be monitored continuously using an HR sensor, and the Borg rating of perceived exertion will be applied 10 seconds before each load change.
3. A cooldown and recovery phase of 5 minutes of active cooldown followed by passive recovery with the participant seated (Figure 1).
4. Guidelines and organization will include controlled temperature and humidity (21-24 °C and 40%-60%, respectively) and previous hydration with water.

### Group 2: PG

**Figure 2:**
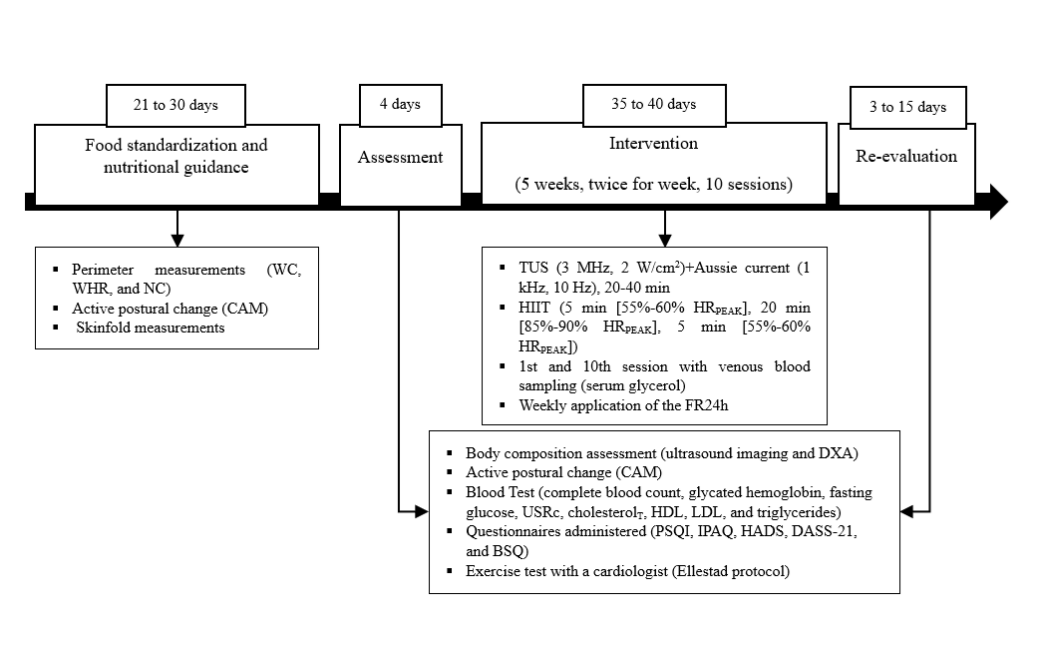
Flowchart of assessments before, during, and after 10 intervention sessions. BSQ: Body Shape Questionnaire; CAM: cardiovascular autonomic modulation; DASS-21: Depression, Anxiety, and Stress Scale; DXA: dual-energy x-ray absorptiometry; FR24h: 24-hour food recall; HADS: Hospital Anxiety and Depression Scale; HDL: high-density lipoprotein; HIIT: high-intensity interval training; HRMAX: maximum heart rate; IPAQ: International Physical Activity Questionnaire (characterization); LDL: low-density lipoprotein; NC: neck circumference; PSQI: Pittsburgh Sleep Quality Index; TUS: therapeutic ultrasound; usCRP: ultrasensitive C-reactive protein; WC: waist circumference; WHR: waist-to-hip ratio.

All the participants in this group will follow the same procedures and sequence as those in the AG. However, the PG participants will receive the TUS and Aussie current treatment in placebo form, ie the therapy switched off. The protocol combines continuous ultrasound (0 MHz; 0 W/cm^2^) and Aussie current (0 kHz; modulated at 0 Hz). Immediately after the TUS+Aussie current treatment, all participants proceed to the treadmill to start the HIIT, following the same protocol as for the AG (Figure 2).

### Group 3: HG

Participants in the HG receive the same treatment as those the AG. However, after the application of TUS plus Aussie current, they will not perform HIIT.

### Outcomes

#### Primary Outcomes

The primary outcomes involve a comparison (between groups) of the effect of 10 sessions of combined therapy (TUS+Aussie current) alone versus in combination with HIIT, specifically on abdominal SAT thickness, body composition, metabolic profile, and serum glycerol release.

#### Secondary Outcomes

Secondary outcomes assess the impact on CAM, sleep quality, anxiety, depression, body image concerns, and blood biochemical variables (Table 2).

**Table 2:** Primary and secondary outcomes of the study.

Variable		Description
**Primary outcomes**		
	Abdominal subcutaneous adipose tissue (cm)	Ultrasound imaging [4,24]
	Body composition (kg; percentage and grams)	Dual-energy x-ray absorptiometry [30]
	Metabolic profile	Venous blood collection Hydrogen nuclear magnetic resonance [34,66]
**Secondary outcomes**		
	Cardiovascular autonomic modulation	Heart rate and arterial pressure variability [36,38,40,41]
	Sleep quality (score)	Pittsburgh Sleep Quality Index [46,47]
	Anxiety, depression, and stress (score)	Hospital Anxiety and Depression Scale [67] Depression, Anxiety, and Stress Scale [48]
	Body image (score)	Body Shape Questionnaire [49]
	Blood tests	Complete blood count, glycated hemoglobin, fasting glucose, ultrasensitive C-reactive protein, total cholesterol, high-density lipoprotein, low-density lipoprotein, and triglycerides
	Perimetry measurements (cm)	Waist-to-hip ratio, waist-to-height ratio, neck circumference, and waist circumference

### Monitoring Adverse Events

There is a possibility that participants may experience side effects in response to the study protocol. Regarding HIIT, intense shortness of breath, generalized tiredness, and dizziness may occur, and this will be recorded in the study. In addition, as the study involves blood sampling, there is a possibility of damage not directly related to the intervention. The expected adverse effects, such as bruising and local pain, will be monitored. Any adverse effects other than these will be recorded and reported, although such occurrences are considered highly unlikely. During assessments and sessions, participants will also be asked about their well-being and whether they have experienced any changes or symptoms since the start of the study. This information will be documented to ensure the ongoing safety and monitoring of the participants.

Participant safety will be prioritized throughout the study. Initially, general health status will be assessed, including a treadmill stress test supervised by a cardiologist to detect cardiovascular contraindications to high-intensity exercise.

A total of 30 days before starting the intervention (TUS+Aussie current), all participants (all 3 groups) will receive identical printed guidelines containing joint mobility and stretching exercises (ankle, hip, and spine) to be performed 2 to 3 times a week. The aim will be to reduce the problems caused by a sedentary lifestyle in the joints and, thus, reduce the risk of injury during HIIT. As a way of monitoring adherence, all participants will complete a weekly form on how often they stretch.

During the HIIT sessions, HR will be monitored continuously, and perceived exertion will be assessed using the Borg scale. Immediate interruption of the session will be planned if signs of intolerance occur (eg, dizziness, chest pain, and syncope). During the application of TUS+Aussie current, participants’ comfort will be monitored using a paresthesia perception scale from 0 to 10, aiming for a level of sensation of approximately 5, with adjustments made whenever necessary. All interventions will strictly follow the safety criteria and contraindications recommended by the equipment manufacturer. In case of adverse events, participants will be clinically assessed and, if necessary, withdrawn from the study to ensure their safety.

### Statistical Analysis

#### Sample Size

The primary outcome, percentage of body fat, was used to calculate the required sample size using the RStudio software (version 12.1; Posit PBC) assuming a moderate expected effect size of *ƒ*≥0.15 and a significance level of 5% and to promote a statistical power of 80%. A total of 18 participants per group is necessary to achieve 80% statistical power with an α level of 5%. Considering a dropout of 10%, the target sample size was increased to 20 participants per group, with the total sample size being 60 individuals. Appropriate statistical corrections (eg, Bonferroni or false discovery rate) will be applied to the *P* values, recognizing the necessity of conducting multiple tests due to the analysis of additional results.

#### Descriptive Statistics and Baseline Data

A CONSORT (Consolidated Standards of Reporting Trials)-style flowchart [68] will present the number of patients selected and all reasons for exclusion before randomization. Demographic and socioeconomic information, including age, sex, race, socioeconomic status, and educational level, as well as baseline clinical characteristics such as BMI, glycated hemoglobin, smoking status, alcohol consumption, and comorbidities, among other variables, will be described by study arm and overall. Categorical variables will be reported as absolute numbers and percentages. Continuous variables with a reasonably symmetrical distribution will be summarized as means and SDs. For highly skewed continuous variables, medians and IQRs will be used.

#### Statistical Methods for Primary and Secondary Outcomes

Data analysis will be conducted by a researcher blinded to the participants’ allocation to the groups. The data will be entered into a Microsoft Excel spreadsheet for subsequent analysis.

For quantitative variables, the assumption of normality of data distribution and homogeneity of variances will be checked using the Shapiro-Wilk test and Levene test, respectively. To compare participant characteristics at baseline between groups, a 1-way ANOVA or Kruskal-Wallis test will be applied when appropriate. For comparisons between groups involving categorical variables, the chi-square test or Fisher exact test will be used. To compare primary and secondary outcomes between and within groups, linear mixed models estimated using the maximum likelihood method with an appropriate covariance matrix structure will be used assuming group (AG, PG, and HG), moment (before and after the intervention) or time (baseline, after TUS+Aussie current, and after HIIT), and interactions (group-moment, group-time, moment-time, and group-moment-time) as fixed factors and participants as a random factor [66]. In addition, moment and time will be assumed as a repeated-measure effect. The model that fits the data will be the one with the minimum Akaike information criterion. When appropriate, the data may be subjected to a Box-Cox transformation to better adhere to a normal distribution [69,70]. Whenever a significant *F* value is obtained in the ANOVA or linear mixed models, a Sidak post hoc adjustment for the purpose of pairwise multiple comparisons will be applied. The significance level will be set at 5% (*P*<.05). All analyses will be conducted using the PASW Statistics software (version 25.0; SPSS).

## Results

The project was registered in the Brazilian Registry of Clinical Trials on February 8, 2023 and updated on December 2, 2024. Currently in the data collection phase. The recruitment and testing phase began in February 2024. Completion of the study is scheduled for the end of September 2025. Data analysis will begin after the last reassessment of this period (September 2025), and the results are expected to be published by 2026.

## Discussion

### Implications

This is the first study to combine TUS+Aussie current with HIIT, potentially integrating the effects of lipolysis and fat oxidation and possible changes in the serum metabolic profile and CAM. The results could optimize treatment duration, promote changes in lipid profile, and maintain cardiovascular health in people with overweight and obesity. The combined protocol is expected to lead to weight loss in the participants compared to the therapies alone. In addition, it is believed that the population with a higher percentage of fat will benefit more from a reduction in abdominal fat thickness (measured through ultrasound imaging) and body fat mass (measured through DXA) after 10 intervention sessions in the AG. This hypothesis assumes that the combination of TUS+Aussie current will promote lipolysis (breakdown of adipocytes measured through serum glycerol collection) and lymphatic drainage into the bloodstream. In addition, by performing HIIT after applying TUS+Aussie current, serum fatty acids will be β-oxidized during exercise.

Scientific literature lacks studies that evaluate the effects of TUS combined with Aussie current with or without HIIT. A controlled, randomized experimental design will be used that follows the SPIRIT guidelines [23] and describes the parameters used to achieve the best results for the population studied.

To the best of our knowledge [4,9,11,15,18], this study will be the first clinical trial to compare the effects of TUS combined with Aussie current and HIIT in participants with overweight and grade-I or grade-II obesity. The findings will provide evidence regarding the effects or lack thereof of this therapeutic approach. Furthermore, as a randomized clinical trial, it will contribute to systematic reviews, fostering new evidence syntheses and addressing existing knowledge gaps.

### Contributions to the Field

This study aims to guide health professionals to design more effective interventions, whether through combined therapy alone or associated with HIIT, for participants with overweight and grade-I or grade-II obesity by determining whether the combination of 2 therapies (TUS+Aussie current and HIIT) is more effective than just one of them in treating or preventing the disease. In addition, the results will contribute to knowledge about a nonpharmacological and noninvasive treatment for obesity and whether the proposed guidelines and interventions will be significantly different in the primary results (reduction in abdominal SAT and change in body composition and blood biochemical variables). In fact, if the results show significant differences between the proposed interventions, patients with obesity will benefit from the combination of 2 noninvasive and fast-acting therapies. In addition, previous studies lasting between 3 and 15 weeks were cited in this study. Thus, this pragmatic study will be able to verify the results of 5 weeks of intervention on the results described [22]. This study describes 10 sessions as this is the treatment protocol offered in most aesthetic clinics.

Previous studies have evaluated the isolated effects of TUS or combined therapies on body composition [4,9,11]; however, important methodological differences exist compared to this study. For example, Fonseca et al [4] investigated the effects of ultrasound in women only using a standardized model for demarcating the abdominal area, not considering individual anatomical variations. In addition, the intervention did not include the use of the Aussie current, and a manual massage protocol was applied after the ultrasound. Similarly, Taha et al [9] combined TUS with aerobic exercise in patients with nonalcoholic fatty liver disease; the exercises consisted of standardized walking sessions (30 minutes) controlled using the Borg scale (12-14), and the time of ultrasound application was not individualized based on abdominal area. Canela et al [11] studied the effects of combined ultrasound and Aussie current therapy associated with whole-body vibration; the sample was restricted to women with cellulite in the gluteal region and did not include a placebo group.

### Prospects for Future Research

While this study will focus on the impact of combining 2 interventions on participants with overweight and grade-I or grade-II obesity, future research could explore comparisons with other exercise protocols, such as high-intensity resistance exercise, and the broader assessment of quality of life. This is particularly relevant given that different exercise modalities elicit distinct musculoskeletal adaptations, which can lead to varied changes in body composition and quality of life.

Although the sequence of TUS+Aussie current is supported by a physiological rationale (ie, the combination of both electrophysical agents aims to optimize fat breakdown and drainage, whereas the subsequent HIIT intervention facilitates β-oxidation of fat), we recognize that the order of the interventions may also influence the effectiveness of this treatment. Therefore, if the combined protocol proves effective, future studies should explore whether reversing the order (ie, applying HIIT before TUS+Aussie current) could lead to different outcomes.

In addition, future research should consider the type and duration of physical training as the total time required for both the TUS+Aussie current intervention and the exercise component may reduce adherence or discourage participation [71]. The choice of HIIT in this protocol is based on its physiological benefits—specifically, its role in promoting β-oxidation and the metabolic use of released fatty acids—while also aiming to optimize the overall time commitment of the intervention.

### Strengths and Weaknesses of This Study

This is a randomized, double-blind, placebo-controlled study designed in accordance with the most rigorous standards for conducting and processing data in clinical trials, ensuring the reliability of the results. It is important to highlight that the intervention period was defined to assess whether the intervention protocol, considering the usual recommendations for exercise and diet, as well as the number of TUS+Aussie current application sessions commonly offered by aesthetic clinics, results in fat reduction for patients with obesity. Thus, this study will provide health care and aesthetic professionals with scientific evidence to understand the mechanisms involved in applying the studied therapies, expected results, and potential systemic impacts. To achieve these objectives, this study includes several outcome measures, including SAT thickness, body composition, metabolic profile, and serum glycerol release, allowing for a comprehensive evaluation of the combined effects of TUS+Aussie current and HIIT. In addition, by incorporating secondary outcomes such as CAM, sleep quality, and perceived stress, this study offers a broader understanding of the physiological and psychological effects of the intervention.

Despite these strengths, this study presents some limitations. The sample size, though adequate for preliminary findings, may not be large enough to detect differences in CAM results. In addition, individual variations in adherence to the HIIT protocol and lifestyle factors such as diet and physical activity outside the study can influence the results. However, it is important to note that strict guidelines are provided to minimize these influences, ensuring greater control over external variables and the reliability of the findings. Furthermore, the study population consists of individuals with overweight and obesity, which may limit the generalizability of the findings to other populations, such as those with severe obesity or metabolic disorders.

In summary, this study will provide a robust framework for evaluating the combined effects of TUS+Aussie current and HIIT on body composition, CAM, and cardiorespiratory and metabolic health. Despite this study’s limitations, the findings will contribute valuable insights into noninvasive strategies for obesity management and may support future research and clinical applications.

## Abbreviations

AG: active therapeutic ultrasound+Aussie current with high-intensity interval training group
AP: arterial pressure
CAM: cardiovascular autonomic modulation
CONSORT: Consolidated Standards of Reporting Trials
DXA: dual-energy x-ray absorptiometry
ECG: electrocardiogram
HG: therapeutic ultrasound+Aussie current–only group
HIIT: high-intensity interval training
HP: heart period
HR: heart rate
LDL: low-density lipoprotein
LF: low frequency
PG: placebo for therapeutic ultrasound+Aussie current with high-intensity interval training group
SAP: systolic arterial pressure
SAT: subcutaneous adipose tissue
SPIRIT: Standard Protocol Items: Recommendations for Interventional Trials
TUS: therapeutic ultrasound
UFSCar: Federal University of São Carlos

## Data Availability

Data sharing is not applicable to this article as no data sets were generated or analyzed during this study. Additional information, including the dataset, statistical code, written consent form, and detailed protocol information, will be made available upon request to the corresponding author following the study’s completion. The data will be made available after the study has been published.
